# Essential…but also vulnerable? Work intensification, effort/reward imbalance, fatigue and psychological health of Spanish cargo drivers during the COVID-19 pandemic

**DOI:** 10.7717/peerj.13050

**Published:** 2022-03-08

**Authors:** Luis Montoro, Boris Cendales, Francisco Alonso, Adela Gonzalez-Marin, Ignacio Lijarcio, Javier Llamazares, Sergio A. Useche

**Affiliations:** 1 Research Institute on Traffic and Road Safety (INTRAS), Universidad de Valencia, Valencia, Spain; 2 Faculty of Economic and Administrative Sciences, El Bosque University, Bogotá, Colombia; 3 Department of Economic and Legal Sciences, University Center of Defense, Santiago del la Ribera, Spain; 4 Department of Technology, ESIC Business and Marketing School, Pozuelo de Alarcón, Community of Madrid, Spain; 5 Spanish Foundation for Road Safety, Madrid, Community of Madrid, Spain

**Keywords:** Professional drivers, Spain, Job stress, Fatigue, Working conditions, Work intensification, Psychological health, COVID-19

## Abstract

**Objective:**

This study investigates the combined effect of the Effort/Reward Imbalance (ERI) model of stress and work intensification within the context of the COVID-19 pandemic on the psychological health (general and work-related fatigue, and psychological strain) of cargo drivers, one of the most demanded workforces during the first year of this pandemic.

**Methods:**

For this cross-sectional research, the data provided by *n* = 1,013 professional drivers from the different 17 autonomous communities (regions) of Spain were analyzed. Participants answered a questionnaire composed of the short version of the Effort Reward Imbalance (ERI) questionnaire, a Work Intensification Scale (WIS) designed for this study, the fatigue subscale of the Checklist Individual Strength (CIS), the Need for Recovery after Work Scale (NFR), and the General Health Questionnaire (GHQ).

**Results:**

Hierarchical regression analyses show that both (ERI and work intensification) models significantly predict driver’s fatigue and psychological strain. The effect of work intensification exists above and beyond the effect of effort/reward imbalance, which has been previously related to the safety performance of cargo drivers.

**Conclusions:**

These findings suggest that the ERI and work intensification models can be complementarily used, especially in scenarios introducing substantial changes in the work environment, such as the COVID-19 crisis. Also, the results of this study support the need to intervene in the working conditions of professional drivers in order to improve their psychological health and well-being during both pandemic and post-pandemic times, as crisis-related management interventions are necessary to promote health and safety in professional drivers in potentially similar contexts in the future.

## Introduction

While the social isolation policies for controlling the COVID-19 pandemic have paralyzed numerous economic activities around the world, some sectors, such as cargo transportation, have intensified their labor in order to meet the increased demand for essential products such as food, cleaning products and medical supplies (Lemke, Apostolopoulos & Sönmez, 2020; The Lancet, 2020). For instance, Spain has been known to be one of the European countries with the highest COVID-19 contagion rate, in a way that the government has suspended various times (*e.g*., during the first lockdown) the 6 hours per day limit of occupational driving in order to facilitate the land circulation of cargo (Ministry of Transport, Mobility & Urban Agenda, 2020). Furthermore, the lack of available personnel has forced drivers to assume physically demanding tasks such as loading and unloading merchandise, and the closure of gas stations and rest areas has drastically restricted access to basic services such as food, bathroom and rest (TransporteProfesionales, 2020).

Beyond objective task-related working conditions, occupational (road) safety of professional drivers has been substantially threatened during the COVID-19 crisis, and key figures support this. While 2020 was one of the years with the fewest fatalities on roads in the recent history of Spain (DGT, 2020), during some months (*e.g*., the first wave of COVID-19), Spanish cargo driver’s fatalities increased by 140%, compared with the average fatality rate reported for the same time interval during the years 2015–2019 (DGT, 2020).

Additionally, it is known that professional drivers are an occupational group that is highly exposed to stressful working conditions, especially work overtime, shift work, low decision latitude, high attentional and ergonomic demands (Gómez et al., 2018; Tse, Flin & Mearns, 2006, 2007; Useche et al., 2021a, Useche, Gómez & Cendales, 2017). In turn, these occupational exposures are associated with physical health problems such as cardiovascular disease, metabolic disorders, obesity, musculoskeletal and gastrointestinal diseases (Cendales, Useche & Gómez, 2014; Đinđić et al., 2013; Imran & Devi, 2013; Kompier & Di Martino, 1995), and negative psychological health outcomes such as anxiety, depression and fatigue (Cendales, Useche & Gómez, 2014; Kompier & Di Martino, 1995; Tse, Flin & Mearns, 2007).

Similarly, stressful working conditions represent a serious safety risk in the transport industry. There is evidence that some psychosocial risk factors at work, such as job strain (high demands and low job control) and effort(high)–rewards(low) imbalance, are associated with risky driving behaviors and work traffic accidents among professional drivers (Kontogiannis, 2006; Useche et al., 2018; Useche, Gómez & Cendales, 2017). Likewise, stress-related health outcomes such as psychological strain and fatigue are also associated with increased risk for traffic crashes (Apostolopoulos, Lemke & Sönmez, 2014; Taylor & Dorn, 2006; Wise, Heaton & Patrician, 2019).

Taking into account the typical occupational exposures of the transportation industry and the increase in job demands caused by the COVID-19 pandemic, it is relevant to examine whether work stressors and the acute intensification of work have a combined effect on the health of professional drivers. This study operationalizes work stress through the Effort–Reward Imbalance (ERI) model (Siegrist, 2002). The ERI model defines work stress as the result of a non-reciprocal social exchange process in which the worker receives a reward for the effort invested in his/her job (salary, esteem, or promotion opportunities) that does not meet his/her expectations (Siegrist & Wahrendorf, 2016). This failed reciprocity experience activates psychophysiological stress reactions (*e.g*., activation in hypothalamic-pituitary-adrenal axis and autonomic nervous system; Coronado et al., 2018; Siegrist & Li, 2017), which in turn are associated with negative physical (Parati & Esler, 2012; Malpas, 2010; Miller, Chen & Zhou, 2007; Lundberg, 2005; Carney, Freedland & Veith, 2005; Lovallo & Gerin, 2003; Goldstein & McEwen, 2002; Esler & Kaye, 2000) and mental health outcomes (Vreeburg et al., 2009; Ströhle & Holsboer, 2003; Friedman & Thayer, 1998; Thayer, Friedman & Borkovec, 1996; Veith et al., 1994; Jacobson & Sapolsky, 1991).

Previous research on professional drivers have associated the ERI model with high blood pressure (Cendales, Useche & Gómez, 2014), fatigue (Tse, Flin & Mearns, 2007; Useche, Gómez & Cendales, 2017), psychological strain (Useche et al., 2021a; Gómez et al., 2018) and increased risk for work traffic accidents (Useche et al., 2020, 2021b). On the other hand, work intensification occurs when the amount of effort that an employee needs to make during the working day increases, in terms of accelerated work pace, simultaneous tasks and reduced idle time (Kubicek, Paškvan & Korunka, 2015).

### The (emerging) problem of work intensification in occupational health research

The adverse effect of work intensification on health is caused by work efforts that have a cumulative “cost” (allostatic load), which in case of insufficient recovery can lead to exhaustion, demotivation and stress-related disease (Bakker & Demerouti, 2007). Recent evidence associates organizational changes that involve work intensification with negative outcomes such as high psychological strain and burnout (Lawrence, Loi & Gudex, 2019; Fein, Skinner & Machin, 2017; Paškvan et al., 2016; Bamberger et al., 2015; Boxall & Macky, 2014), reduced home-work balance (Fein, Skinner & Machin, 2017; Boxall & Macky, 2014) and low job satisfaction (Paškvan et al., 2016; Kubicek, Paškvan & Korunka, 2015; Boxall & Macky, 2014).

At this time, there are no studies that combine the perspectives of the ERI model and work intensification. The complementarity of these perspectives lies in that while the ERI model defines stress from the perspective of an exchange contract between employees and employer, work intensification specifically focuses on changes in the amount of work to be done in a fixed time. In other words, work intensification represents an additional stressor (possibly transient, in this case) to high effort—low rewards working conditions, and therefore an additional risk to workers’ health.

Thus, this research extends the knowledge about psychosocial risk factors at work by examining whether work intensification and the ERI model have a combined effect on professional drivers’ health, and whether the effect of work intensification on health exists above and beyond the effect of effort-reward imbalance.

### Objective and hypotheses of the study

This study aimed to assess the combined effect of the Effort/Reward Imbalance (ERI) model of stress and work intensification within the context of the COVID-19 pandemic on the psychological health (*i.e*., general and work-related fatigue, and psychological strain) of Spanish cargo drivers. In this regard, and bearing in mind the previous evidence appended in the introduction, it was hypothesized that:

Hypothesis 1: Effort/Reward Imbalance positively explains psychological health problems among Spanish cargo drivers.
Hypothesis 2: Work intensification during COVID-19 pandemic positively explains psychological health problems in freight drivers, above and beyond the variance explained by the ERI model of stress.

In practical terms, the results of this study may suggest lines of intervention to mitigate the consequences of work intensification in an occupational group with a high risk of stress-related disease (professional drivers). Furthermore, these interventions are urgent in the current post-pandemic context, as the mental health problems generated by the COVID-19 pandemic may increase the economic and social cost of this public health emergency (Holmes et al., 2020).

### Understanding key work psychosocial concepts: a summary

For this study, fatigue and psychological strain (which are indicators of psychological health) were used as criterion variables. The association of these psychological health outcomes with working conditions is widely documented in the general population (Rugulies, Aust & Madsen, 2016, 2017; Dobryakova et al., 2013; Fahlén et al., 2006; Stansfeld & Candy, 2006; Van Vegchel et al., 2005) and in professional drivers (Gómez et al., 2018; Useche, Gómez & Cendales, 2017; Cendales, Useche & Gómez, 2014; Tse, Flin & Mearns, 2007).

Chronic fatigue is characterized by reduced patterns of ineffective action, reduced interest, involvement, commitment, concentration and motivation, which can manifest both at work and in other life domains (Bültmann et al., 2000). When fatigue is specifically related to work, it is known as the need for recovery (Van Veldhoven & Broersen, 2003). In the professional driving context, fatigue can manifest with feelings of tiredness, sleepiness or loss of vigilant attention due to prolonged exposure to extended driving periods, as well as monotonous or difficult road conditions (Kee, Mohd & Goh, 2010; Boksem, Meijman & Lorist, 2005). Numerous studies on road safety highlight fatigue as one of the main risk factors for traffic accidents (Moradi, Nazari & Rahmani, 2019; Dawson et al., 2018; Davidović, Pešić & Antić, 2018; Alaiakbari & Moridpour, 2017).

Finally, the psychological strain is defined as disruptions in the normal functioning of a person, and the recent appearance of distressing psychosomatic symptoms (Goldberg, 1978; Shevlin & Adamson, 2005). As it has been mentioned, it is known that psychological strain and fatigue problems represent a high burden of disease in the occupational group of professional drivers. At the same time, they are associated with negative safety outcomes such as risky driving behaviors and traffic accidents (Wise, Heaton & Patrician, 2019; Apostolopoulos, Lemke & Sönmez, 2014; Taylor & Dorn, 2006).

## Materials and Methods

### Participants

The sample of this study is composed of 1,013 cargo drivers from different autonomous communities of Spain. As this is an overrepresented workforce in terms of gender, 97.6% of partakers were men. The participants’ average age was *M* = 48.5 years (*SD* = 7.9).

On average, professional drivers composing the sample had a job tenure (at their present workplaces) of *M* = 17.3 years (*SD* = 10.8), driving for *M* = 8.1 h a day (*SD* = 1.5), for *M* = 5.0 days a week (*SD* = 0.6). Only 28% of the participants reported doing shift work during the first 12 months of the pandemic, compared to 27% already working under this modality before COVID-19.

### Research design, procedure and ethics

For this cross-sectional study, participants were contacted through the National Federation of Transport Associations of Spain (FENADISMER). Given the current situation, the study questionnaire was sent and completed in an electronic (online) format during the year 2020, in which professional driving was considered one of the few “essential” occupations across all the national territory. The inclusion criteria for this study were: (*i*) being a cargo driver working in Spain, and (*ii*) having been active at their jobs during the emergency.

In order to achieve an acceptable degree of representativeness, the entire Spanish census of cargo drivers (about 390,000 drivers for the year 2020, according to the Federation) was assumed as population size. Although population representativeness can be only partially assumed on the basis of a non-probabilistic sampling method, an *a priori* calculation of the minimum sample size was carried out using the following formula:

(1) $$(S = {z^2}\sigma/{e^2})$$

where S represents the sample size; *z* the standardized value for a 95% level of confidence (*z* = 1.96), σ represents the standard deviation (commonly set as 0.50), and *e* represents the maximum error allowed, with α = 0.05_(5%)_. The resulting number suggested a minimum of about 769 subjects. The participation rate was about 60%, as approximately 1,800 invitations were initially sent.

No personal data that could allow for the identification of participants was collected. All drivers partaking in the study sample provided their informed consent, certifying their agreement before responding to the online questionnaire. The research protocol was reviewed and approved by the Research Ethics Committee of the Research Institute on Traffic and Road Safety (INTRAS)–Institutional Review Board at the University of Valencia (IRB number HE0001190920).

### Statistical analysis (data processing)

After careful data curation, descriptive data (*i.e*., means, standard deviations and errors) were calculated, and questionnaire-related study variables were scored in accordance with the guidelines provided by each instrument. The bivariate associations between study variables were assessed by means of Spearman (*r*_*s*_) correlations, considering their robustness over Pearson (*r*) coefficients when ordinal values are measured (Liu et al., 2016).

In regard to factor analyses, and as this was a new scale developed for this study, the factorial structure of the WIS was assessed through Exploratory and Confirmatory Factor Analyses (EFA and CFA, respectively) and sequentially tested. The EFAs (exploratory analyses) used a maximum likelihood (ML) method with Promax oblique rotations (item-based factor loadings are available in Table 1). On the other hand, the CFA (confirmatory analyses), apart from factor loadings, used goodness-of-fit (GOF) criteria based on indexes from different families and logics, following the criteria proposed by many sources: in these regards, CFI/NFI/TLI/IFI indexes over 0.94; an RMSEA under 0.06; and a CMIN/DF < 5.00 can be considered as suitable indicators of GOF (Hu & Bentler, 1999; Useche et al., 2021b).

**Table 1:** Reliability statistics of the work Intensification during the COVID-19 crisis Scale (WIS). The first column presents the correlation of each item with the WIS scale, while the reliability coefficients (if each element is removed) are appended at the right one.

Item	λ	Correlation with the total scale	Cronbach’s Alpha if the element is deleted
I drive during more hours than before the COVID-19 crisis	0.684	0.532	0.768
I have more time pressure than before the COVID-19 crisis	0.704	0.562	0.763
Driving during the COVID-19 crisis, I have more distractions than before	0.651	0.508	0.771
During the COVID-19 crisis I have been forced to exceed the speed limit more than before	0.698	0.545	0.767
When I travel, I hardly find places where I can eat, sleep or rest	0.427	0.372	0.796
During the COVID-19 crisis I have been forced to do loading/unloading without help	0.593	0.474	0.779
During the COVID-19 crisis I am more afraid of having a traffic accident	0.685	0.557	0.763
During the COVID-19 crisis I’m drinking coffee or stimulants, so that I don’t fall asleep when driving	0.681	0.530	0.768

With the aim of exploring the relationships among study variables in a directional approach, and testing the study hypotheses, hierarchical linear regressions were used. These regressions examined the combined effect of the ERI and work intensification models on psychological health (general fatigue, need for recovery and psychological strain). As the participants are from different cargo transport companies and variability in their working conditions was expected, the daily hours and days of the week spent working were introduced during the first step of the regression models. In the second step, the effort/reward imbalance score was added, and, in the third step, the work intensification score. The effect of the predictors on the criterion variables was defined according to the standardized regression coefficients and to the change in R^2^ for each step of the regression models. All statistical analyses were carried out using the Statistical Package for Social Sciences (IBM SPSS©), version 26.0 (2020).

### Instruments

The electronic form (questionnaire) was composed of a battery of instruments addressing various psychosocial spheres. For this purpose, the authors received permission to use this battery of instruments from the copyright holders. The different sections of the questionnaire are described below:

*Efforts-Reward imbalance*: a short version of the Efforts-Reward imbalance scale (Siegrist, 2002), previously validated by Useche et al. (2021a) in transportation workers was used. The *efforts* (*example of item:* “I have constant time pressure due to a heavy workload”) and *rewards* (*example of item:* “I receive the respect I deserve from my superiors”; Useche, Gómez & Cendales, 2017) have 3 (α = 0.75) and 7 (α = 0.79) Likert-scaled items. All items are scored in a 4-point rating scale ranging from 1 (strongly disagree) to 4 (strongly agree). Scores of *Efforts* and *Rewards* were calculated by averaging the items of each subscale, and the effort-reward imbalance was the ratio (Effort/Rewards) between these two subscales. The full contents of this version of the ERI and further technical insights provided by the scale are available in its validation paper (Useche et al., 2020).

*General fatigue*: It was measured by using the fatigue subscale of the Checklist Individual Strength (CIS; Vercoulen et al., 1994), which consists of eight items (*e.g*., “I feel tired”) responded on a 7-point Likert scale, where 1 = “Yes, it is true” and 7 = “No, that is not true”. The Cronbach’s alpha coefficient of the scale was α = 0.81. The general fatigue score was calculated by averaging all the CIS items. In addition, The Need for Recovery after Work Scale (NFR; Sluiter et al., 2003) was used to measure work-related fatigue. The NFR consists of 11 dichotomous items (Yes/No) that investigate the presence of symptoms (*e.g*., “I find it difficult to relax at the end of a working day”) of fatigue during the previous month. The Cronbach’s alpha coefficient of the scale was α = 0.87. The need for recovery score was calculated by adding up all the NFR items.

*Psychological strain:* The General Health Questionnaire (GHQ-12; Goldberg, 1992) was used to measure psychological strain. The GHQ-12 (α = 0.82) investigates disruptions in normal functions and the recent appearance of distressing psychosomatic symptoms using a 4 level Likert scale where 1 means “none at all” and 4 “a lot more than usual”. A general score of psychological “strain” or “distress” can be calculated by averaging the GHQ-12 items, after recoding the negative questions.

*Work Intensification*: It was measured through an 8-item scale (WIS; α = 0.80) designed specifically for this research. The Likert-based scale ranging (0–4) measures work intensification retrospectively by asking whether professional drivers perceive an increase in their quantitative work demands from the moment the COVID-19 crisis was officially recognized by the Spanish government (March 14th, 2020), and whether the workload of professional cargo drivers increased, as they were considered an “essential workforce” for keeping supply chains in a country level.

This scale is similar to the questionnaire proposed by Kubicek, Paškvan & Korunka (2015), which was designed for non-specific work intensification situations, but evaluates perceived changes in the work organization caused by a specific event and during a shorter time window. In contrast, the WIS scale refers to specific conditions commonly observable in the cargo transport industry during the COVID-19 pandemic.

Table 1 shows the WIS item composition, their factor loads (from Exploratory Factor Analysis–EFA), the items’ correlation with the total scale, and the Cronbach’s Alpha if the item is deleted (all item λs > 0.40; 42.75% of variance explained). These results endorsed overall the unifactorial structure of the scale, without the need of excluding any item from the proposed questionnaire.

With the aim of corroborating the adequacy of the scale structure as suggested by the exploratory analyses, a Confirmatory Factor Analysis (CFA) was subsequently performed, whose outcomes endorsed these assumptions, with: CFI = 0.984; NFI = 0.978; TLI = 0.966; IFI = 0.984; RMSEA = 0.051; CMIN/df = 3.61), thus suggesting that the items for this scale can be optimally grouped in a single dimension.

## Results

### Descriptive statistics

Table 2 shows the descriptive statistics of the study variables. Participants reported high levels of effort/reward imbalance (scores higher than 1.0 imply an increased risk for stress-related disease). On average, work intensification was relatively low. Effort, effort/reward imbalance and work intensification were positively and significantly associated with all indicators of psychological health (*i.e*., general fatigue, need for recovery and psychological strain).

**Table 2:** Descriptive statistics and bivariate correlations of study variables. Each correlation represents the association between two variables; the first is described in the left column (variable names), paired with the second at each row (numbered in accordance to each column). Correlations with asterisks can be understood as statistically significant.

Variable	Mean	SD	2	3	4	5	6	7	8	9	10	11
1. Age	48.49	7.87	0.441**	−0.094**	0.026	−0.059	−0.016	−0.046	−0.100**	−0.042	−0.057	−0.104**
2. Job tenure	17.30	1.83		−0.092**	−0.042	−0.089**	0.061	−0.101**	−0.039	−0.044	−0.025	−0.019
3. Days driving (week)	5.26	0.62			0.123**	0.032	−0.049	0.030	0.068*	−0.017	0.037	0.046
4. Hours driving (day)	8.11	1.51				−0.023	0.055	−0.043	0.033	−0.001	0.026	−0.035
5. ERI-Effort	2.19	0.71					−0.050	0.844**	0.544**	0.388**	0.416**	0.238**
6. ERI-Rewards	2.49	0.42						−0.510**	0.093**	−0.039	−0.068*	−0.026
7. E/R Imbalance	2.13	0.84							0.383**	0.347**	0.383**	0.217**
8. Work intensification	1.82	0.79								0.489**	0.465**	0.383**
9. General Fatigue	4.18	1.47									0.727**	0.595**
10. Need for Recovery	5.91	3.15										0.559**
11. Psychological strain	22.85	5.76										

**Notes:**

* Correlation is significant at the level *p* < 0.050.

** Correlation is significant at the level *p* < 0.010.

On the other hand, ERI’s rewards were only negatively and significantly associated with the need for recovery. Associations of work intensification with psychological health indicators were stronger than those of the ERI model. Age, job tenure, days worked (week), and hours worked (day) were not significantly associated with any indicator of psychological health.

Table 3 summarizes the hierarchical linear regression models used to predict the professional drivers’ psychological health indicators. In conjunction, the predictors introduced in the models significantly explained 27.2% (*F* = 75.385, *p* < 0.001) of general fatigue, 26.7% (*F* = 73.291, *p* < 0.001) of need for recovery and 19.0% (*F* = 47.325, *p* < 0.001) of psychological strain.

**Table 3:** Hierarchical linear regression models to predict professional drivers’ psychological health indicators. This table appends the variables included in the significant hierarchical regression model (HRM) used to predict cargo drivers’ psychological health indicators. A total of three steps were used to achieve the final model presented in the table.

Predictors	General fatigue		Need for recovery		Psychological strain	
	Beta	∆R^2^	Beta	∆R^2^	Beta	∆R^2^
*Step 1*						
Age	−0.106 (*p* = 0.205)	0.011 (*p* = 0.010)	−0.053 (*p* = 0.087)	0.019 (*p* = 0.000)	−0.106 (*p* = 0.001)	0.020 (*p* = 0.000)
Hours driving (day)	0.038 (*p* = 0.212)		0.101 (*p* = 0.010)		0.038 (*p* = 0.329)	
Days driving (week)	−0.112 (*p* = 0.129)		0.034 (*p* = 0.387)		−0.112 (*p* = 0.004)	
*Step 2*						
E/R Imbalance	0.258 (*p* < 0.000)	0.110 (*p* = 0.000)	0.381 (*p* = 0.000)	0.132 (*p* = 0.000)	0.258 (*p* = 0.000)	0.061 (*p* = 0.000)
*Step 3*						
Work intensification	0.364 (*p* = 0.000)	0.151 (*p* = 0.000)	0.375 (*p* = 0.000)	0.117 (*p* = 0.000)	0.364 (*p* = 0.000)	0.110 (*p* = 0.000)
Adjusted R^2^	0.268		0.262		0.188	

According to Hypothesis 1, after controlling the effects of age, days working a week and hours working a day, effort/reward imbalance was significantly associated with all indicators of psychological health. Furthermore, and regarding Hypothesis 2, work intensification explained the variance of all criterion variables above and beyond the variance explained by the ERI model.

## Discussion and conclusion

This study investigated the combined effect of effort/reward imbalance and work intensification caused by the COVID-19 crisis on the psychological health of cargo drivers. As expected, it was found that both models (ERI and work intensification) significantly predict psychological health, and that the effect of work intensification exists above and beyond the effect of effort/reward imbalance.

At first glance, these results are consistent with the existing evidence on the ERI model (Gómez et al., 2018; Useche, Gómez & Cendales, 2017; Rugulies, Aust & Madsen, 2016, 2017; Cendales, Useche & Gómez, 2014; Dobryakova et al., 2013; Stansfeld & Candy, 2006; Tse, Flin & Mearns, 2007; Fahlén et al., 2006; Van Vegchel et al., 2005), and with studies that define work intensification as a stressor that operationally differs from stable working conditions, thus introducing unexpected dynamics, efforts and demands to the work environment (Bamberger et al., 2015; Boxall & Macky, 2014; Fein, Skinner & Machin, 2017; Kubicek, Paškvan & Korunka, 2015; Lawrence, Loi & Gudex, 2019; Paškvan et al., 2016).

In theoretical terms, the findings of this study suggest that the ERI and work intensification models (using the WIS scale) can be considered as complementary approaches that, when used together, they might improve the evaluation of psychosocial risk factors at work in times like the current COVID-19 pandemic, where, apart from constituting an unforeseen issue, work intensification had a considerable degree among the addressed workforce (Ministry of Transport, Mobility & Urban Agenda, 2020).

One of the facts beyond this rationale is that the ERI and WIS contents, far from being incompatible or redundant, might result in complementary ways to address both work contents and changes: on the one hand, the ERI questionnaire addresses structural features of the workplace through an timeless approach, thus allowing to measure usual working conditions and perceptions. On the other, the WIS contents address changes due to the impact of the ‘denaturalization’ of the typical working conditions (in this case because of the pandemic), regardless of their baseline values or perceptions reported in these regards.

As for further psychosocial health-related risks, the results of this study are also in line with the evidence that points to professional drivers as an occupational group with high effort/reward imbalance and increased risk for stress-related disease (Gómez et al., 2018; Coronado et al., 2018; Cendales, Useche & Gómez, 2014; Tse, Flin & Mearns, 2007; Useche, Gómez & Cendales, 2017). A first insight over it is the fact that the effect of work intensification on the psychological health of cargo drivers is statistically significant and has a considerable magnitude, even stronger than the effect of the ERI indicator itself. This theoretically remarkable issue suggests that, although only scarcely explored in the empirical literature, work intensification might constitute a key issue to consider in further occupational health studies dealing with this highly vulnerable population, especially in scenarios of increased job demands, even considering that the latter is typically characterized as high in previous literature (Davidović, Pešić & Antić, 2018; Gómez et al., 2018; Đinđić et al., 2013).

Following an applied approach, the findings of this study support the urgency of evaluating and intervening in psychosocial work risk factors of professional drivers. To date, most of the interventions focused on stress at work in this occupational group have an individual approach, based on coping skills training, as well as health and road safety education (Lemke & Apostolopoulos, 2015; Ng et al., 2015; Puhkala et al., 2016; Pylkkönen et al., 2013). Although, in general, these individual interventions have proven to be effective, it is necessary to complement them with changes in work organization. In particular, managing fatigue can be very difficult if training is not combined with changes in working conditions (May & Baldwin, 2009).

The need for these “cocktail” interventions (directed simultaneously to the work organization and to the workers) is justified if approached from the view that work stress among professional drivers (or any other workforce) is a multi-causal phenomenon, “for which there are no single and simple solutions” (Kompier et al., 2000). For example, the organization of work shifts and rest periods can be even more effective in reducing stress-related outcomes such as fatigue if they are implemented in combination with the promotion of healthy habits. This same approach could be applied to the few existing interventions that are focused on organization-level variables of professional driving, such as driving schedule, social support, the mechanical condition and ergonomic design of vehicles, and driving technological assistance (Lemke, Apostolopoulos & Sönmez, 2020; Hatami et al., 2019; Lemke & Apostolopoulos, 2015; Ng et al., 2015; Apostolopoulos, Lemke & Sönmez, 2014).

In an evaluation of 13 natural experiments, Kompier et al. (2000) found that commitment of management and key employees, good flow of information, rigorous, participatory, interdisciplinary and long-term oriented organization can be stimulating factors for interventions in the transportation industry. On the other hand, factors such as external management, conflicts between employees and resistance to change can obstruct the effectiveness of stress management interventions. Age and workload, which in this study were factors associated with psychological health, should also be taken into account to tailor occupational health programs to the characteristics of each population of drivers.

In addition, considering the associations between occupational stress, fatigue and the risk for work traffic accidents (Wise, Heaton & Patrician, 2019; Apostolopoulos, Lemke & Sönmez, 2014; Taylor & Dorn, 2006), high-quality occupational health programs may help manage problems such as the increase in accidents on inter-municipal roads in Spain during the COVID-19 pandemic (National Observatory of Road Safety, 2020).

### Limitations of the study and further research

Although the sample size used for this study was relatively extensive, and the basic parameters for statistical procedures were successfully met, some key limitations are to acknowledge. Firstly, the cross-sectional design, implying that all the variables were measured simultaneously and predictive relationships over time cannot be fully established, prevents the authors from making causality attributions. Instead, the findings must be interpreted in the light of a set of associations among variables that, although statistically significant, cannot be verified without (*e.g*.) longitudinal or test-retest approaches (Begg, Langley & Williams, 1999; Boo & Choi, 2021).

Secondly, the exclusive use of self-report measures prevents us from determining whether assessing psychosocial risks at work reflects objective working conditions or subjective perceptions of workers. In the particular case of work intensification, the evaluation of organizational change could suggest the need to use longitudinal methods. However, there is evidence that workers can reliably report changes in job demands, even over long periods of time (Kubicek, Paškvan & Korunka, 2015).

Likewise, the high reliability of the used instruments compensates for possible self-reporting biases, especially when it is considered that the topic addressed was considerably sensitive for all the population during the emergency state. In any case, prolonged monitoring of changes in work organization associated with COVID-19 can extend the findings of this study and contribute to the investigation of the social and economic burden of the COVID-19 pandemic.

Further, our study raised interesting insights on conjunctural workplace-related issues also remarked in other studies, such as the high geographical mobility of cargo drivers and the limited access they had to protective materials needed for preventing the transmission of infectious diseases (Lemke, Apostolopoulos & Sönmez, 2020; The Lancet, 2020). Besides psychosocial factors at work such as stress and fatigue, whose role in driver’s occupational safety is still undisputable, these issues could be analyzed in depth to determine to what extent they could contribute to impair safety and health of cargo drivers. This is especially valuable if it is considered that understanding and intervening in the work environment of transportation workers in difficult situations such as the COVID-19 crisis with a sound empirical basis (*i.e*., learning from experience), may constitute a necessary first step for promoting their safety in a similar future context.

Finally, it is worth remarking the need to further analyse the relationships between work intensification and professional drivers’ actual behavioral and safety-related outcomes, including (*e.g*.) driving behavioral questionnaire data, near-misses, and work traffic crash reports.

## Supplemental Information

10.7717/peerj.13050/supp-1 Supplemental Information 1 Raw Data. The scores of each of the items used during the research process.

## Additional Information and Declarations

### Data Availability

The following information was supplied regarding data availability: The raw data are available in the Supplemental File.
